# Habitat Preferences and Growth of *Ruditapes bruguieri* (Bivalvia: Veneridae) at the Northern Boundary of Its Range

**DOI:** 10.1155/2014/235416

**Published:** 2014-01-16

**Authors:** Alla V. Silina

**Affiliations:** A.V. Zhirmunsky Institute of Marine Biology, Far East Branch of the Russian Academy of Sciences, Vladivostok 690059, Russia

## Abstract

This work is the first attempt to study growth and some morphological parameters of the clam *Ruditapes bruguieri*, as well as its habitat preferences. It is found that *R. bruguieri* lives on bottom sediments including pebble and coarse- and medium-grained sand with a slight admixture of silt. At the study area, this bivalve inhabits sea waters with good aeration, stable oceanic water salinity, and a high oxygen concentration. The annual fluctuations of the water temperature from 13-14°C (in winter) to 22–29°C (in summer) are close to the threshold temperature values, within which the species can exist. Near the boundary of the species range, along the Jeju Island coasts, south of Republic of Korea, 83.8% of all clams die during the coldest period of the year. Here, annual rings are formed on *R. bruguieri* shells during winter. The maximum age of *R. bruguieri*, determined during the study, is 6.5 years, but the clam samples contain mainly individuals at 3.0–3.5 years of age (34%). The largest clam dimensions are 36.0 × 26.5 mm (length × height of shell). At the study area, a usual shell length is 20.0–32.0 mm (75% of all the collected individuals).

## 1. Introduction

The marine Veneridae, which is the largest family of Bivalvia and one of the least studied and most poorly defined molluscan taxa, comprises approximately 800 species, including some of the most economically important and abundant ones [1]. Two species of the genus *Ruditapes* (Bivalvia: Veneroida: Veneridae) are found along the coasts of Korea: the Manila clam *Ruditapes philippinarum* (Adams et Reeve, 1848) and the clam *Ruditapes bruguieri* (Hanley, 1845) [2–5]. The commercial species *R. philippinarum*, including its populations in coastal waters of Korea, is considered in a variety of papers published, but studies on *R. bruguieri* are few in number, despite the latter species is widely spread in the Indo-West Pacific. According to OBIS Indo-Pacific Molluscan Database, 2006-05-17, the synonyms of mollusk *R. bruguieri *are as follows: *Paphia (Amygdala) bruguieri auct*.; *Venus bruguieri* (Hanley, 1845); *Tapes tessellata* (Adams and Reeve), 1850; *Tapes variegata* (Sowerby, 1852); *Ruditapes variegata* (Sowerby, 1852); *Tapes violacens* (Deshayes, 1853); *Tapes japonica* (Deshayes, 1853); *Tapes cinerea* (Deshayes, 1854); *Tapes punicea* (Deshayes, 1854); and *Tapes semidecussata* (Reeve, 1864). There are also such synonyms as *Tapes bruguieri, Venerupis (Ruditapes) bruguieri*, and *Venerupis bruguieri*.

*R. bruguieri* is a tropical-subtropical species [2, 4]. It is distributed in the tropical Indo-West Pacific: Central and East Indian Ocean (West India and East India, Andhra Pradesh, Thailand, Andaman, and Nicobar), Indo-Malaysia (northwestern, northern, and northeastern coasts of Australia, Philippines, Indonesia, and Oceania), Papua New Guinea, China (South China Sea, including Hong Kong and East China Sea), and Japan [6–9]. It also occurs in the Temperate West Pacific of the Northern Hemisphere: South Korea, Korea Bay, and North China [2–5]. Huber [10] believes that the range of *R. bruguieri* is limited to the Indian Ocean. From Japan and Australia, only *Ruditapes aspera* (Quoy and Gaimard, 1835) is known, whereas both species occur in waters of India.

Little is known about the biology and ecology of *R. bruguieri*. There are no data on growth of this bivalve species. Therefore, the present study investigates some ecological preferences and growth of *R. bruguieri*. Usually, surveys of populations inhabiting marginal areas of the species range are of particular interest for the study of biological and ecological characteristics of the species as in such cases it is possible to reveal threshold values of some environmental parameters that restrict the area of the species natural habitat. For instance, a sea water temperature at the northern boundary of species areal can indicate the thresholds of tolerant temperature limits for the species. As for the range of the tropical-subtropical *R. bruguieri*, its northern boundary runs particularly along the coasts of Korea, and, for this reason, the samples of *R. bruguieri* from the coastal waters of Jeju Island, South Korea, were studied in the work. Besides, a comparative analysis of ecological parameters of Jeju Island's bays, where *R. bruguieri* was or was not found by the author and other researchers, has been conducted with the use of the literature data to reveal ecological preferences of the species. Growth and morphometric data for the species were obtained using the clam shell samples from the littoral zones of Jeju Island, as it is known that samples from shell assemblages may be used for growth rate estimation of clams, and such samples give a more comprehensive appreciation of the maximum size and life span of the species than the sample of live mollusks from the natural habitat [11].

## 2. Materials and Methods

### 2.1. Ecological Characteristics of Sites of Clam Collections

Jeju Island is a volcanic formation located about 80 km south of the mainland. Its coastline is mainly rocky with several sandy and pebble beaches. The warm Tsushima Current flowing from the southwest creates a subtropical climate on the island with an average annual water temperature of about 15°C [2, 4, 12]. In the studied area, the water salinity is normal, usually oceanic [12, 13]. The island is subject to moderate tides and strong wave action [4].

**Figure 1:**
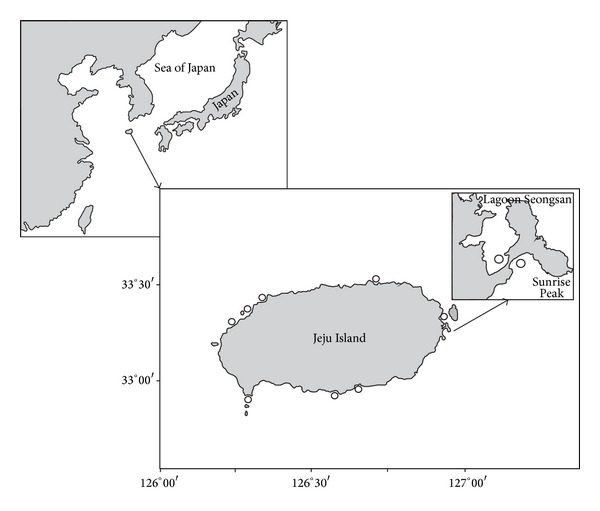
Sites of searches for *Ruditapes bruguieri *shells along the coasts of Jeju Island, South Korea.

Searches for clam *R. bruguieri* shells that were lying on the beach were carried out in the littoral zone (in the upper-tide and mid-tide levels) of several bays situated on the northwestern, southern, and eastern coasts of Jeju Island during low tides in 2008 (Figure 1). The sampling efforts were similar for all sites. Everywhere, the shells were collected from the area of about 350 m^2^ (70 m × 5 m). Some of clam shell samples from the littoral zone of the northern, northwestern, southern, and eastern coasts of the island were kindly provided by K. A. Lutaenko (A.V. Zhirmunsky Institute of Marine Biology, FEB RAS (IMB FEB RAS)). The latter samples were collected in 2007, 2008, 2011, and 2012. These clam shells were also collected from the upper-tide and mid-tide levels of the littoral zones during low tides.

On the eastern coast of Jeju Island, clam shells were looked for in two bays near Ojo-ri town. The littoral zone was examined both in the protected Seongsan lagoon (33°27′27.18′′N, 126°56′00.39′′E) and in the open bay (33°27′30.47′′N, 126°56′05.49′′E) at the cape with Sunrise Peak volcano (Figure 1). The lagoon is almost flat and shallow, protected from wave impacts by the cape. The bottom sediments of the lagoon are composed of sand with various grain size, pebble, and small boulders [14]. Here, shells of the clam *R. bruguieri* were not found. Silt constitutes 1% of the weight of dry soft sediments [15]. In the open bay, the littoral bottom sediments consist of middle-grained sand between volcanic reefs (basalt tectonic plates). Silt makes up 2% of the weight of dry soft sediments [15]. Here, a total of 117 clam shells were collected in 2007, 2008, and 2012. Also, at the open northern area of the beach at Jongdal-ri town (33°30′19′′N, 126°54′44′′E), 11 clam shells were sampled in 2011.

The open southern site was on Namo Beach (33°12′34.82′′N, 126°15′45.64′′E). Here, the littoral coastal bottom sediments consist of grey middle- and fine-grained sand in the upper-tide and mid-tide level, passing into silt (>63% of the weight of dry sediments) in the low-tide level [14, 15]. Only two clam shells were found in 2008. Also, on the open rocky Yerae coast, approximately 8 km west of Seogwipo city (33°14′24′′N, 126°23′45′′E), 11 clam shells were collected in 2011 and 2012.

One northwestern site was situated on Geumneung Beach (Hallim Park) (33°23′23.07′′N, 126°13′48.38′′E), opposite to small Biyangdo Island. It is an open bay with the coastal bottom sediments composed of light coarse- and middle-grained sand with small admixture of silt (about 0.1%) among big volcanic boulders [14, 15]. Other sites on the northwestern coast of Jeju Island were situated on Kwakji Beach (33°27′05′′N 126°18′21′′E) and Hyupchae Beach (33°23′35′′N 126°14′14′′E). On the northwestern coasts, 12 clam shells were collected in 2008 and 2011.

Near the northern (N-NE, Figure 1) coast of Jeju Island, *R. bruguieri* shells were sampled from organogenic sand of Hamdeok Beach (25 km southwest of Aewol town). Here, 12 clam shells were found in 2008.

### 2.2. Clam Characteristics and Statistical Analysis

Shell length (*L*, mm, the longest distance between the anterior and posterior shell edges), shell height (*H*, mm), and shell width (*D*, mm, if both valves were available) were measured using sliding calipers to an accuracy of 0.1 mm. Age and growth rates of each individual were determined retrospectively by annual growth rings on the outer surface of the shell. As clam shell samples were not numerous, for subsequent comparison of growth rates, the samples were combined into four united samples (northwestern, northern, eastern, and southern samples) coast according to the geographic location of the samples (Figure 1, Table 1).

The data were expressed as mean values ± standard error of the mean (SE). Prior to statistical analysis, all data were tested for normality of variance among the different groups by using a Kolmogorov-Smirnov test. The method of linear regression was used to reveal relationships between morphometric parameters of shell, as well as variations in the length/height ratio with age of mollusk. A Student's *t*-test was used to identify significant differences among mean shell heights for scallops of the same age at different sites. Data processing was done using STATISTICA, version 5.1, and Microsoft Office Excel, Data Analysis.

## 3. Results and Discussion

### 3.1. Habitat Preferences

#### 3.1.1. Bottom Sediments

The largest in number samples of *R. bruguieri* shells were collected on the middle- and coarse-grained sand and sand/gravel beaches on the open eastern coast of the island. However, shells of the studied bivalve species were almost absent on bottom sediments with a high content of fine-grained sand and silt at the southern site, Namo Beach. Earlier, shells of *R. bruguieri* were collected on the western littoral zone of small Seogundo Island, situated nearby Jeju Island [4]. Seogundo Island has a mainly rocky coastline with small sand and gravel areas [2]. Here, shells were also found on coarse-grained sand. Thus, these data can be sign for a conclusion that *R. bruguieri* prefers coarse- and middle-grained sand with a slight admixture of silt. The silt content in the bottom sediments may constitute 1-2%. It indicates that the species is, most likely, sensitive to mud resuspension in the water.

#### 3.1.2. Water Aeration, Salinity, and Oxygen Concentration

Shells of the clam *R. bruguieri* were found in the open bays exposed to strong wave action. Here, the clam habitats are washed only by oceanic waters. At the eastern site, situated in the protected Seongsan lagoon, shells of this species were not found despite the bottom sediments that were suitable for the clam.

As is known, the water salinity at the coasts of Jeju Island is 33.0–34.9‰. The oxygen concentration in the water is high, 7.29–8.14 m L^−1^ [16, 17]. The chlorophyll concentration ranges from 0.71 to 1.71 mg L^−1^ (mean ± SE, 1.11 ± 0.2 mg L^−1^). The concentrations of nitrate, phosphate, and silicate are 0.029–0.206 mg L^−1^ (0.101 ± 0.03 mg L^−1^), 0.001–0.027 mg L^−1^ (0.007 ± 0.004 mg L^−1^), and 0.024–0.682 mg L^−1^ (0.454 ± 0.2 mg L^−1^), respectively [16, 17]. This can be sign that* R. bruguieri* prefers habitats with good water aeration, stable oceanic water salinity, and high oxygen concentration.

#### 3.1.3. Water Temperature

All the samples of *R. bruguieri* from the coasts of Jeju Island, collected by the author and other researchers, were not large in number. This is an evidence of sparseness of the *R. bruguieri* populations in these waters. This is typical for populations inhabiting marginal areas of the species range. Jeju Island is situated at the northern boundary of *R. bruguieri *range. In cases like this, it is just the winter water temperature that restricts vital functions of animal and determines the lower threshold of temperature favorable for the species. The minimum temperature, 13-14°C, is observed in January and February along the coasts of Jeju Island [2, 4, 12]. Therefore, a water temperature of about 13-14°C is close to the lower limit of favorable temperatures for *R. bruguieri*. In summer, the water temperature at the coasts of the Island is about of 22–26°C with the maximum in August [2, 4, 12]. At the southern coast of the island, surface water temperature varies from 14.5°C in March to 29.5°C in August; at the depth of 20 m, the water temperature ranges from 14.2°C in March to 27.2°C in September [16, 17]. Thus, the annual fluctuations of the water temperature from 13-14°C to 22–29°C can be considered close to the temperature range, within which the studied bivalve species can exist.

### 3.2. Growth Rates and Relationships between Morphometric Parameters

The mean value of the ratio of shell length (*L*, mm) to shell height (*H*, mm) was 1.47 ± 0.00, which means that shell is elongated. This ratio was not found to vary with age; that is, the shape of shell does not change as mollusk grows, and the shell proportions remain almost constant during its lifecycle:

(1) $$\begin{array}{c} \frac{L}{H}=-0.0003L+1.4732,\;\;R=0.0009,\;\;N=154. \end{array}$$

The regression describing the relationships between the length, height, and width (*D*, mm) of* R. bruguieri* shells revealed that these parameters correlate positively at a high degree of confidence:

(2) L=1.3929H+1.0951,  R  =  0.98,  N=154,H=0.6904L−0.1341,  R=0.98,  N=154,D=0.4296L−0.8671,  R=0.96,  N=77.

**Figure 2:**
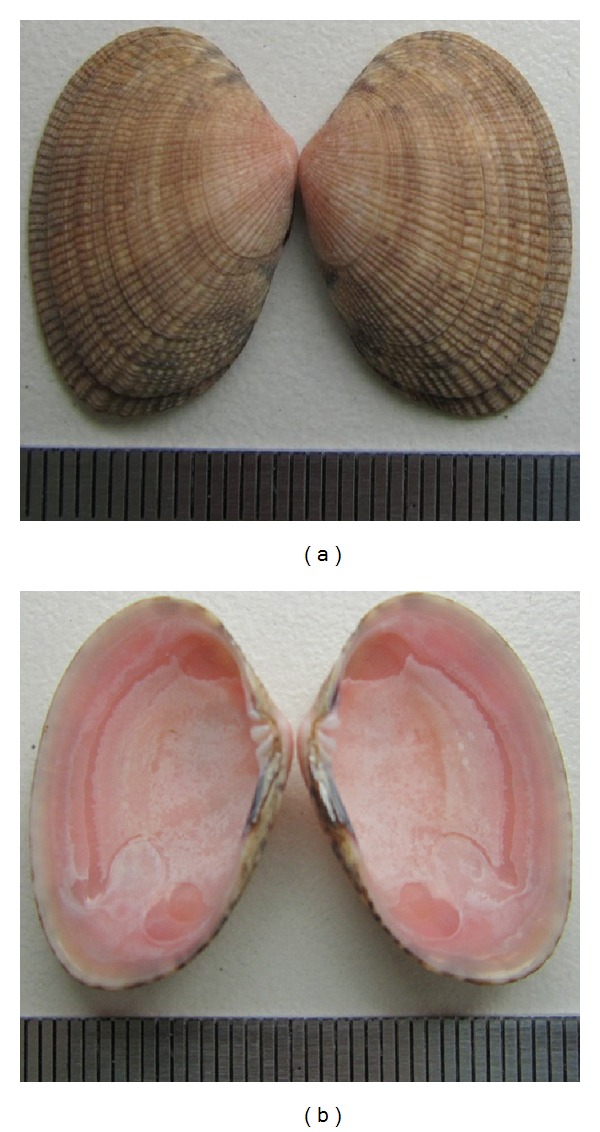
The external and inner surfaces of *Ruditapes bruguieri* shell.

It is known that Manila clam *R. philippinarum*, which is closely related to *R. bruguieri*, forms annual winter rings on the outer surface of its shell [18]. Shells of *R. bruguieri* have rings similar to those of *R. philippinarum* (Figure 2). It is logical to suppose that *R. bruguieri* also forms growth ring during the cold winter period, taking into account that Jeju Island is situated near the northern boundary of *R. bruguieri *range and retardation in its shell growth is most probable just in winter. Moreover, it is known that warm-water bivalves, which usually form annual rings on their shells during the hottest period of the year in the middle and southern portions of their natural habitat, form annual rings in the coldest period at the northern boundary of their range, for example, *Anadara *(*Scapharca*)* broughtoni* [19]. In late October, two clams with remains of soft tissues were found; that is, they had died shortly before. Near the shell edge, each specimen had almost complete annual increment (from the last ring to the shell edge). New shell ring at the shell edge was not formed. Therefore, the number of rings on the outer surface of *R. bruguieri* shell was used in this work to estimate age of mollusks, and the distance from shell apex to each ring was measured to determine their growth rates retrospectively.

*R. bruguieri* formed broad annual shell increments, especially during the first three years of life, that enabled to determine the season when a mollusk died by the structure near the shell edge. Thus, the season of death was determined for each individual. Formed winter rings at the edges were found in 83.8% of shells. This indicates that the main portion of individuals perished during the coldest period of the year. Only 11.7% of individuals died during summer and 4.5% during the other seasons. This is the expected result for such tropical-subtropical species as *R. bruguieri* inhabiting the coastal waters of Jeju Island, which are near the northern boundary of the range of this mollusk species.

The obtained data on growth of* R. bruguieri* showed a high variability of this process in the studied area (Table 1). It is partly explained by the interannual fluctuations of environmental parameters, as the specimens were from different age cohorts. As a rule, scattering of the morphometric indices is more than usual for organisms inhabiting areas near boundaries of their species range, where one or several environmental parameters are close to the threshold values for the species tolerance and especially affect its growth rate. A Student's *t*-test did not reveal significant differences (at a level of 99%) among mean shell heights for scallops of the same age at different sites. At a level of 95%, the differences were revealed between the shell heights of the clams collected from the northwestern and southern coasts (shells with 4 and 5 annual rings), clams sampled from the northwestern and eastern coasts (shells with 4 and 5 annual rings), and clams sampled from the northern and eastern coasts (shells with 3 annual rings). It is known that the sea water at the southern coast of Jeju Island is warmer than the one at the northern coast due to the warm Tsushima Current flowing from the southwest [4]. Most likely, this is the reason of higher clam growth rates at the south than at the north (Table 1).

**Figure 3:**
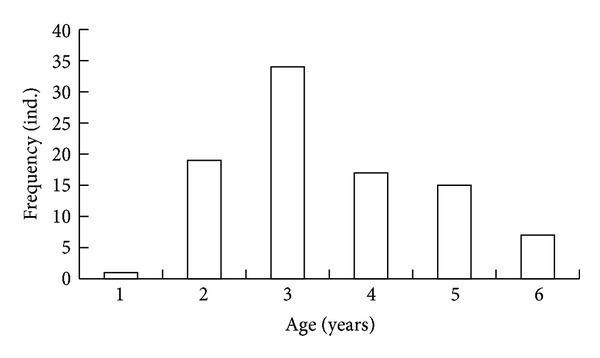
Age distribution of the *Ruditapes bruguieri* sample from the eastern coast of Jeju Island.

The *R. bruguieri* samples from the coasts of Jeju Island included individuals at 0.5–6.5 years of age. The maximum age of *R. bruguieri* was 6.5 years. At the eastern coast of Jeju Island, the age of mollusks in the sample varied within 0.6–5.5 years, mostly 3.0–3.5 years (34%) (Figure 3).

**Figure 4:**
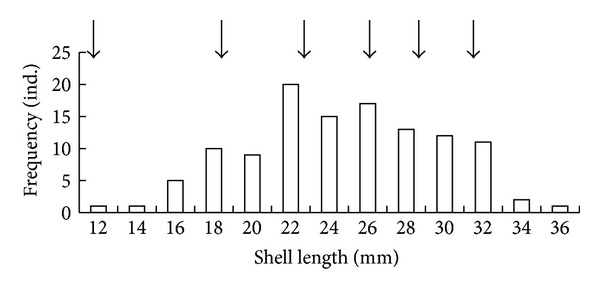
Distribution of shell lengths in the *Ruditapes bruguieri* sample from the eastern coast of Jeju Island. Arrows indicate the shell lengths of clams with 1, 2, 3, 4, 5, and 6 annual winter rings, respectively.

Length, height, and width ranges of *R. bruguieri* shells sampled along the Jeju Island coasts were 8.0–36.0 mm (mean value 23.6 ± 0.4 mm), 5.0–26.5 mm (16.2 ± 0.3 mm), and 3.6–13.5 mm (9.0 ± 0.2 mm), respectively. At the eastern coast of Jeju Island, the sample consisted of individuals with the shell length of 8.0–36.0 mm, mainly 20.0–32.0 mm (75%) (Figure 4). The peaks in the histogram of shell length distribution corresponded to the values of shell length of individuals with 2, 3, and 4 annual rings (indicated by arrows in Figure 4); the heights of these peaks correlated with the heights of the peaks in the histogram of age distribution. The peaks for 5- and 6-year-old individuals did not stand out, as the difference between values of the shell lengths of 5- and 6-year olds was small. These findings were an additional evidence of the annual periodicity of shell ring formation.

## 4. Conclusion

Thus, the obtained data can be sign for a conclusion that *R. bruguieri* prefers coarse- and middle-grained sand and gravel with a slight admixture of silt. At the study area, this bivalve inhabits the sites with good water aeration, stable oceanic water salinity, 33.0–34.4‰, and a high oxygen concentration, 7.29–8.14 mg L^−1^. As the winter water temperature is about 13-14°C near Jeju Island, at northern boundary of* R. bruguieri* range, it indicates that this water temperature is close to the lower limit of favorable temperature for this mollusk. The maximum summer water temperature at the Island is about of 22–29°C. Along the coasts of Jeju Island, South Korea, which is near the boundary of the species range, 83.8% of all individuals die during the coldest period of the year. Here, annual rings are formed on *R. bruguieri* shells during the winter. Near the boundary of the species range, the maximum age of *R. bruguieri*, determined during the study, is 6.5 years, but the clam samples mainly consist of 3.0–3.5-year-old individuals (34%). The maximum dimensions of clams are 36.0 × 26.5 mm (length × height of shell). At the study area, a usual shell length is 20.0–32.0 mm (75% of all individuals).

## Figures and Tables

**Table 1:** Size limits and growth rates of the clam *Ruditapes  bruguieri* along the Jeju Island coast, South Korea. The mean values ± standard error of the mean and min–max limits for shell parameters are shown.

Area	*N*	Shell size, mm		Shell height at the respective annual ring, mm				
		Length	Height	1	2	3	4	5
Northern coast	12	18.7 ± 1.6 10.0–29.0	13.4 ± 1.2 6.8–21.0	6.8 ± 0.1	11.5 ± 0.3	14.5 ± 0.3	17.1 ± 0.1	19.4 ± 0.2
Northwestern coast	12	22.8 ± 2.0 8.0–33.0	15.8 ± 1.4 5.0–24.0	6.5 ± 0.4	11.3 ± 0.5	14.5 ± 0.5	16.4 ± 0.5	18.4 ± 0.9
Southern coast	13	25.4 ± 1.4 18.5–36.0	17.5 ± 1.0 12.5–26.0	7.0 ± 0.4	11.5 ± 0.4	14.9 ± 0.5	17.7 ± 0.6	20.0 ± 1.0
Eastern coast	117	24.0 ± 0.4 13.3–35.5	16.4 ± 0.4 9.0–35.5	6.4 ± 0.1	11.8 ± 0.2	15.1 ± 0.2	17.4 ± 0.2	19.3 ± 0.2
